# A Selective Cyclic Peptidic Human SIRT5 Inhibitor

**DOI:** 10.3390/molecules21091217

**Published:** 2016-09-10

**Authors:** Jiajia Liu, Yajun Huang, Weiping Zheng

**Affiliations:** School of Pharmacy, Jiangsu University, 301 Xuefu Road, Zhenjiang 212013, Jiangsu Province, China; liujiajia2401@163.com (J.L.); huangyajun0205@163.com (Y.H.)

**Keywords:** sirtuin, SIRT5, inhibitor, cyclic peptide, *N*^ε^-carboxyethyl-thiocarbamoyl-lysine

## Abstract

In the current study, we discovered that a side chain-to-side chain cyclic pentapeptide harboring a central *N*^ε^-carboxyethyl-thiocarbamoyl-lysine residue behaved as a strong and selective (versus human SIRT1/2/3/6) inhibitor against human SIRT5-catalyzed deacylation reaction. This compound was also found to be proteolytically much more stable than its linear counterpart. This compound could be a valuable lead for developing stronger, selective, metabolically stable, and cell permeable human SIRT5 inhibitors.

## 1. Introduction

The evolutionarily-conserved sirtuin family of enzymes refers to a group of β-nicotinamide adenine dinucleotide (β-NAD^+^)-dependent protein *N*^ε^-acyl-lysine deacylases catalyzing a deacylation reaction that serves as one way of reversing the post-translational lysine side chain *N*^ε^-acylation [1,2,3,4,5]. Sirtuin family members are present in organisms from all the three life forms, and seven members (i.e., SIRT1-7) have been known to be present in mammals, including humans [5].

**Figure 1:**
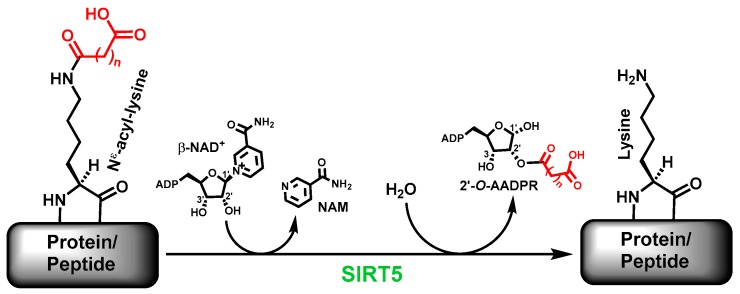
The SIRT5-catalyzed β-NAD^+^-dependent *N*^ε^-acyl-lysine deacylation reactions, including demalonylation (*n* = 1), desuccinylation (*n* = 2), and deglutarylation (*n* = 3). Since these three deacylase activities of SIRT5 are stronger than its deacetylase activity, this latter enzymatic activity is not depicted here. ADP, adenosine diphosphate; β-NAD^+^, β-nicotinamide adenine dinucleotide; NAM, nicotinamide; and 2’-*O-*AADPR, 2’-*O-*acyl-ADP-ribose.

The sirtuin-catalyzed deacylation includes the removal of the simple acetyl group and the bulkier acyl groups on specific *N*^ε^-acyl-lysine residues on proteins [1,2]. Many sirtuins are now known to more proficiently catalyze the removal of acyl groups bulkier than acetyl; human SIRT5 is one of such sirtuins. Specifically, the major deacylase activity of human SIRT5 is to catalyze the removal of the malonyl, succinyl, and glutaryl groups, respectively, from *N*^ε^-malonyl-lysine, *N*^ε^-succinyl-lysine, and *N*^ε^-glutaryl-lysine (Figure 1) [6,7,8,9]. Of note, besides the deacylated product, a sirtuin-catalyzed deacylation reaction also generates another two products, i.e., nicotinamide (NAM) and 2’-*O*-acyl-ADP-ribose (2’-*O*-AADPR) [1].

The sirtuin-catalyzed deacylation reaction plays an important role in regulating such crucial cellular processes as transcription, DNA damage repair, and metabolism [10,11,12], and is regarded as a contemporary therapeutic target for cancer, and metabolic/neurodegenerative diseases [13,14,15,16]. As a sirtuin family member present in both cytosol and mitochondrial matrix [17], SIRT5 is intimately involved in the metabolic regulation in these two cellular compartments, according to current studies [8,9,18,19]. In order to further define the functional roles (including mechanistic details) and the therapeutic potentials of the SIRT5-catalyzed deacylation, its potent and selective chemical modulators would be desirable so that the observed functional/pharmacological consequences following the treatment with such modulators could be more confidently correlated with the SIRT5-catalyzed deacylation.

**Figure 2:**
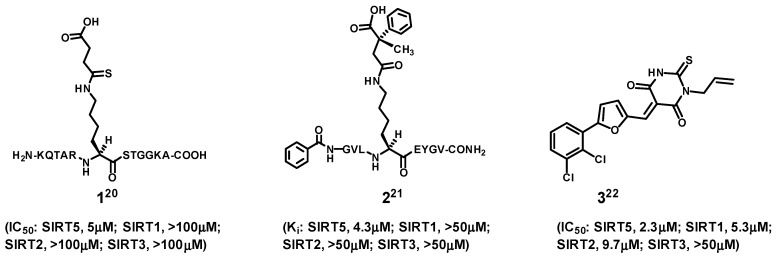
The chemical structures of the three notable examples of human SIRT5 inhibitors (i.e., compounds **1**–**3**) reported in the current literature, and their inhibitory potencies (IC_50_ or K_i_ values) against SIRT5, SIRT1, SIRT2, and SIRT3, respectively. Note: all of the IC_50_ or K_i_ values were obtained in SIRT5 desuccinylation assay.

In the area of SIRT5 inhibitor development, compounds **1**–**3** shown in Figure 2 represent the three notable examples reported in the current literature [20,21,22]. Among them, compounds **1** and **2** are the strongest and most selective SIRT5 inhibitors. However, their being linear peptides would render them less ideal as molecular entities useful as potential therapeutic agents and research tools, because of the metabolic instability and membrane impermeability generally associated with linear peptides [23].

## 2. Results and Discussion

**Figure 3:**
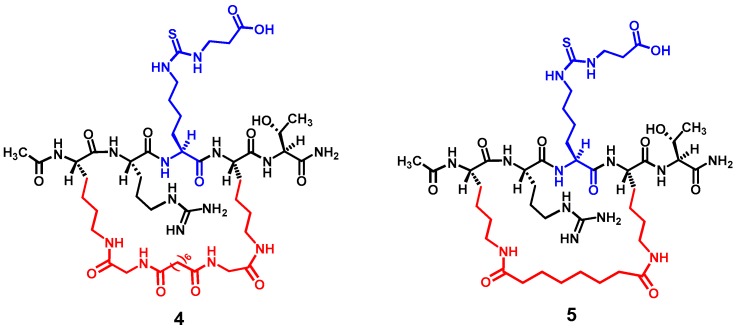
The chemical structures of the cyclic peptides **4** and **5** designed in the current study. Macrocyclic bridging units are shown in red, and the central warhead residue is shown in blue.

Considering that peptide macrocyclization is an established approach to conferring upon a linear peptide an enhanced metabolic stability and cell permeability [23], we endeavored in the current study to see if potent and selective cyclic peptide-based SIRT5 inhibitor(s) could be identified. Figure 3 depicts the two designed cyclic peptides (**4** and **5**). One salient feature of these molecules is their harboring a central N^ε^-carboxyethyl-thiocarbamoyl-lysine residue that was previously identified in our laboratory to be a strong and selective (versus SIRT1 and SIRT6) thiourea-type catalytic mechanism-based SIRT5 inhibitory warhead [24]. It should be noted that compound **1** depicted in Figure 2 also harbors a catalytic mechanism-based SIRT5 inhibitory warhead (i.e., *N*^ε^-thiosuccinyl-lysine) [20], however, due to the concern over potential cellular toxicity resulting from the use of the thioamide-containing *N*^ε^-thioacyl-lysine type of warheads [25,26,27,28,29], we opt to use the thiourea-type warhead *N*^ε^-carboxyethyl-thiocarbamoyl-lysine to potentially circumvent the cytotoxicity issue. However, the central five-residue fragment of compound **1** (i.e., AR-(*N*^ε^-thiosuccinyl-lysine)-ST) was used as the basis in the current study to construct the cyclic pentapeptides **4** and **5**. This is due to the previous demonstrations that the binding interactions of a *N*^ε^-acetyl-lysine-containing peptide substrate with a sirtuin active site are predominantly mediated by *N*^ε^-acetyl-lysine itself and its four immediately flanking amino acid residues (two on each side) [30,31], and that a catalytic mechanism-based sirtuin inhibitor is first recognized and processed by a sirtuin enzyme as an alternate substrate [1,32].

**Scheme 1:**
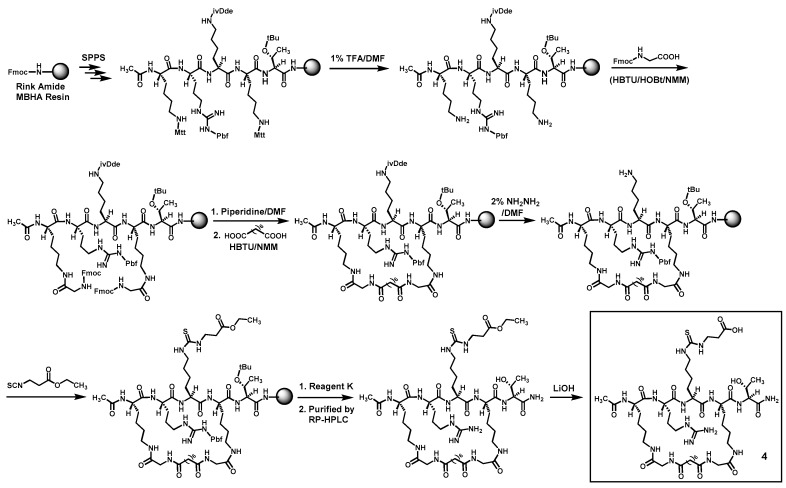
The solid phase synthesis of compound **4**.

**Scheme 2:**
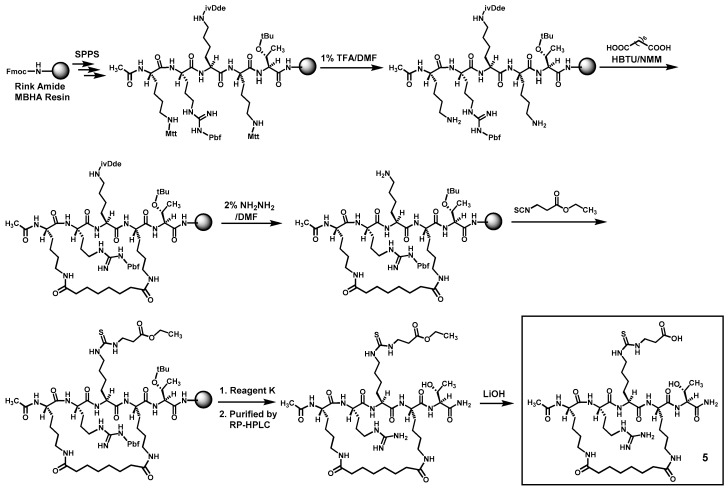
The solid phase synthesis of compound **5**.

Compounds **4** and **5** were prepared according to Scheme 1 and Scheme 2 by the Fmoc chemistry-based manual solid phase peptide synthesis (SPPS) on Rink Amide 4-methylbenzhydrylamine (MBHA) resin. The protecting groups Mtt and ivDde on lysine side chains were orthogonally deprotected respectively with 1% (*v*/*v*) trifluoroacetic acid (TFA)/*N*,*N*-dimethylformamide (DMF) and 2% (*v*/*v*) hydrazine/DMF, as shown. Following the on-resin peptide macrocyclization and formation of the central *N*^ε^-ethoxycarbonylethyl-thiocarbamoyl-lysine residue, a cyclic peptide was cleaved from the resin with a TFA-containing cocktail (Reagent K). The obtained crude cyclic peptide was then purified by semi-preparative reversed-phase high performance liquid chromatography (RP-HPLC). The side chain ethyl ester on the central *N*^ε^-ethoxycarbonylethyl-thiocarbamoyl-lysine residue of thus obtained cyclic peptide was subsequently hydrolyzed in solution with LiOH, affording the crude **4** and **5**, which were again purified with semi-preparative RP-HPLC. The exact masses of the purified **4** and **5** were confirmed by high-resolution mass spectrometry (HRMS) analysis (Table 1).

When compound **4** was prepared and assessed for its ability to inhibit the SIRT5-catalyzed deacylation reaction, we found that it behaved as a SIRT5 inhibitor with an IC_50_ value of ~6 μM (Table 2). Of note, this IC_50_ value is comparable to that for compound **1** determined by Lin and co-workers under a similar SIRT5 deacylation inhibition assay condition [20]. Moreover, compound **4** seems to be a stronger inhibitor against the SIRT5-catalyzed deacylation reaction than the truncated linear pentapeptide of compound **1**, i.e., CH_3_CONH-AR-(*N*^ε^-thiosuccinyl-lysine)-ST-CONH_2_ whose IC_50_ value was determined to be ~40 μM in the same study by Lin and co-workers [20]. Compound **4** was subsequently assessed for its inhibitory power against the deacylation reaction catalyzed by other human sirtuins, and for this, we first checked its inhibitory potency against the SIRT2-catalyzed deacetylation reaction, since SIRT2 was not included in our previous study as an example sirtuin to be assayed with our SIRT5 inhibitory warhead *N*^ε^-carboxyethyl-thiocarbamoyl-lysine [24]. We found that compound **4** exhibited a SIRT2 inhibitory potency (IC_50_ ~9.2 μM) comparable to that against SIRT5 (Table 2). While this finding may imply that *N*^ε^-carboxyethyl-thiocarbamoyl-lysine could also be a strong SIRT2 inhibitory warhead, we would be more inclined to the following interpretation: even though the central *N*^ε^-carboxyethyl-thiocarbamoyl-lysine residue in compound **4** is a selective SIRT5 inhibitory warhead, this selectivity could be overridden by the presence of an appropriate structural architecture immediately surrounding the warhead. Given that our goal is to find cyclic peptide-based potent and selective SIRT5 inhibitor(s), we subsequently prepared compound **5** whose macrocyclic bridging unit is different from that in compound **4**, as shown in Figure 3.

Compound **5** was first assessed for its inhibitory power against the SIRT5-catalyzed deacylation reaction, and as shown in Table 2, it also exhibited a strong SIRT5 inhibition with an IC_50_ value of ~7.5 μM. More importantly, when it was subjected to a SIRT2 assay, we found that this compound was a very weak inhibitor against the SIRT2-catalyzed deacetylation reaction with an IC_50_ value greater than 1 mM (Table 2). The observed differential SIRT5/2 inhibitory profiles of compounds **4** and **5** suggested that the macrocycle bridging unit in compound **4** would somehow help to position this molecule favorably at SIRT2 active site, most likely with its central warhead residue binding at a region away from the *N*^ε^-acetyl-lysine binding tunnel; whereas the macrocycle bridging unit in compound **5** would impose a disfavored binding of this molecule at SIRT2 active site, with its central warhead residue either unable to bind or binding weakly at the *N*^ε^-acetyl-lysine binding tunnel. The outcome would be a diminished (for **4**) or a maximized (for **5**) binding affinity difference at SIRT2 and SIRT5 active sites.

Compound **5** was further evaluated for its inhibitory power against other human sirtuins. As shown in Table 2, it was found to be also a very weak inhibitor against the deacylation reactions respectively catalyzed by human SIRT1, SIRT3, and SIRT6.

**Figure 4:**
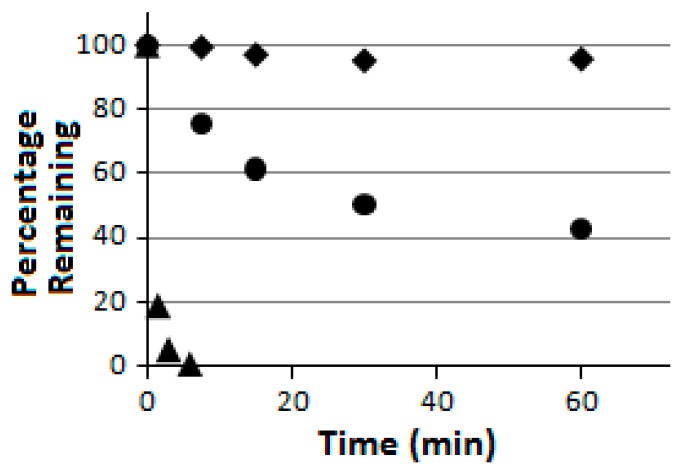
The pronase digestion profiles of compounds **5** (◆), **6** (●), and the linear pentapeptide control H_2_N-HK-(N^ε^-acetyl-lysine)-LM-COOH (▲).

Compound **5** was further subjected to a proteolytic digestion assay using pronase as the peptidase/protease preparation [33]. It was found to be proteolytically much more stable than the linear pentapeptide control H_2_N-HK-(*N*^ε^-acetyl-lysine)-LM-COOH (Figure 4). More importantly, as indicated in Figure 4, compound **5** was also found to be proteolytically much more stable than its linear counterpart **6** which was also prepared in the current study. These findings are consistent with the notion that peptide chain macrocyclization may also lead to an enhanced proteolytic stability [23]. It should be noted that the linear pentapeptide control H_2_N-HK-(*N*^ε^-acetyl-lysine)-LM-COOH is the SIRT1/2/3 substrate used in the current study (see “Experimental Section”), and it has been used as a control in the pronase digestion assay on all occasions in our laboratory to ascertain that the pronase preparation used was active.

**Scheme 3:**
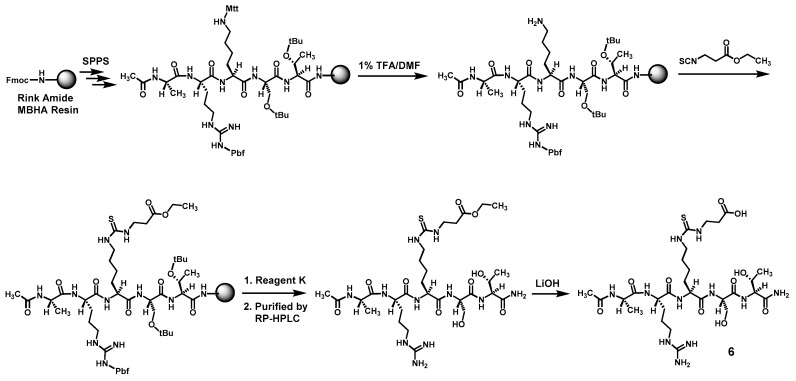
The synthesis of compound **6**.

Compound **6** was prepared according to Scheme 3 also by the Fmoc chemistry-based manual SPPS on Rink Amide MBHA resin. The protecting group Mtt on the central lysine side chain was orthogonally deprotected with 1% (*v*/*v*) TFA/DMF. Following the reaction of the exposed free amino group with ethyl 3-isothiocyanatopropionate with the formation of the central *N*^ε^-ethoxycarbonylethyl-thiocarbamoyl-lysine residue, the pentapeptide intermediate was cleaved from the resin with Reagent K. The obtained crude pentapeptide was then purified by semi-preparative RP-HPLC, and the side chain ethyl ester on its central *N*^ε^-ethoxycarbonylethyl-thiocarbamoyl-lysine residue was subsequently hydrolyzed in solution with LiOH, affording the crude **6** which was again purified with semi-preparative RP-HPLC. The exact mass of the purified **6** was confirmed by a unit-resolution electrospray ionization-mass spectrometry (ESI-MS) analysis: Calcd. *m*/*z* for C_28_H_52_N_11_O_10_S ([M + H]^+^) 734.36; found: 734.90. Calcd. *m*/*z* for C_28_H_53_N_11_O_10_S ([M + 2H]^2+^) 367.68; found: 368.14.

The availability of compound **6** also prompted us to perform a comparative evaluation of the SIRT5 inhibitory power of **6** and its cyclic counterparts **4** and **5**, since peptide chain macrocyclization may also enhance the target binding affinity of a ligand [23]. When testing compound **6** side-by-side with compounds **4** and **5** under our SIRT5 inhibition assay condition, we found that compound **6** exhibited a comparable SIRT5 inhibitory potency to those of compounds **4** and **5** (Table 2), suggesting that the particular macrocyclic bridging units in compounds **4** and **5** were unable to constrain the peptidic backbone of **4** and **5** into a bioactive conformation or were able to interfere with the overall binding of compounds **4** and **5** at SIRT5 active site, or both. This scenario is different from what we observed previously with SIRT1/2/3/6, in which the same macrocyclic bridging units in compounds **4** and **5** were able to confer significantly enhanced inhibitory potency upon a parent linear peptidic inhibitor against SIRT1, 2, 3, or 6 [34,35]. These observations have also further reinforced the notion that sirtuin active site substrate specificity exists [1,32,36]. Compound **6** was further assessed for its inhibitory power against SIRT1/2/3/6. As shown in Table 2, while compound **6** was found to be a very weak inhibitor against SIRT1/3/6, its inhibition against SIRT2 was found to be only about 13-fold weaker than that against SIRT5. This finding further suggested that a *N*^ε^-carboxyethyl-thiocarbamoyl-lysine-containing SIRT5 inhibitor may also exhibit a reasonable SIRT2 inhibition with its power contingent upon the identity of the structural architecture immediately surrounding the SIRT5 inhibitory warhead *N*^ε^-carboxyethyl-thiocarbamoyl-lysine.

## 3. Experimental Section

### 3.1. General

The following materials were purchased from commercial vendors for the compound preparation, and were used as received without further treatment. Sigma-Aldrich China (Shanghai, China): *N*-methylmorpholine (NMM), trifluoroacetic acid (TFA), *N*,*N*-dimethylformamide (DMF), hydrazine; TCI Shanghai (Shanghai, China): suberic acid; Alfa Aesar China (Shanghai, China): 2-(1H-benzotriazole-1-yl)-1,1,3,3-tetramethylaminium hexafluorophosphate (HBTU), *N*-hydroxybenzotriazole (HOBt), phenol, thioanisole, ethanedithiol, ethyl 3-isothiocyanatopropionate, LiOH; Honeywell China (Shanghai, China): acetonitrile, methanol; Sinopharm Chemical Reagent Co., Ltd. (Shanghai, China): piperidine, acetic anhydride, diethyl ether; Shanghai Plus Bio-Sci & Tech Co., Ltd. (Shanghai, China): Rink Amide MBHA resin (loading capacity = 0.34 mmol/g).

The N^α^-Fmoc-protected amino acids were purchased from TCI Shanghai, GL Biochem (Shanghai) Ltd. (Shanghai, China), or Shanghai Plus Bio-Sci and Tech Co., Ltd.

Routine unit-resolution mass spectrometry was performed on a Thermo LXQ LC-ion trap mass spectrometer (Thermo Scientific, Waltham, MA, USA) at Jiangsu University. High-resolution mass spectrometry (HRMS) was performed on an AB 5600+ Q TOF high-resolution mass spectrometer (AB Sciex LLC, Framingham, MA, USA) at the Pharmacy School of Fudan University.

The following materials were purchased from commercial vendors for the sirtuin inhibition assay and the pronase digestion assay, and were used as received without further treatment. Sigma-Aldrich China: the active human recombinant His_6_-SIRT1, Trizma, Hepes, β-NAD^+^, a 1.0 M solution of MgCl_2_ (molecular-biology grade), the pronase from *Streptomyces griseus*; Cayman Chemical (Ann Arbor, Michigan, USA): the active human recombinant GST-SIRT1, the active human recombinant His_6_-SIRT2, the active human recombinant His_6_-SIRT3, the active human recombinant GST-SIRT5, the active human recombinant His_6_-SIRT6; TCI Shanghai: d,l-dithiothreitol (DTT); Alfa Aesar China: NaCl, KCl.

The peptide substrates prepared and used in the sirtuin inhibition assay were: the SIRT1/2/3 substrate H_2_N-HK-[*N*^ε^-acetyl-lysine]-LM-COOH corresponding to amino acids 380–384 of the human p53 protein acetylated at K^382^; the SIRT5 substrate CH_3_CONH-AR-[*N*^ε^-succinyl-lysine]-ST-CONH_2_ corresponding to amino acids 7–11 of the human histone H3 protein succinylated at K^9^; the SIRT6 substrate H_2_N-EALPK-[*N*^ε^-myristoyl-lysine]-TGGPQ-CONH_2_ corresponding to amino acids 15–25 of the human tumor necrosis factor α (TNFα) myristoylated at K^20^.

### 3.2. Synthesis of ***4*** (Scheme 1)

This compound was prepared by the Fmoc chemistry-based manual SPPS on Rink Amide MBHA resin. For each amino acid coupling reaction, four equivalents of a N^α^-Fmoc-protected amino acid, 3.8 equivalents of the coupling reagent HBTU and the additive HOBt were used in the presence of 0.4 M NMM/DMF, and the coupling reaction was allowed to proceed at room temperature for 1 h. A 20% (*v*/*v*) piperidine/DMF solution was used for Fmoc removal. After the completion of the on-resin amino acid assembling and the N-terminal α-amino group acetylation with acetic anhydride in the presence of 0.4 M NMM/DMF, the side chain Mtt protecting group from two Lys(Mtt) residues were selectively removed with a 1% (*v*/*v*) TFA/DMF solution before the two exposed free amino groups were acylated with Fmoc-glycine under peptide coupling reaction condition. After the Fmoc removal from the two incorporated Fmoc-glycine residues with a 20% (*v*/*v*) piperidine/DMF solution, the two newly exposed free amino groups were then acylated with suberic acid at room temperature for 1 h under peptide coupling reaction condition. The resulting resin-bound cyclized peptide was then treated with a 2% (*v*/*v*) solution of hydrazine (NH_2_NH_2_) in DMF, the exposed free amino group at the central position was then reacted with ethyl 3-isothiocyanatopropionate (2 × 5 h). The subsequent treatment with reagent K (83.6% (*v*/*v*) TFA, 5.9% (*v*/*v*) phenol, 4.2% (*v*/*v*) ddH_2_O, 4.2% (*v*/*v*) thioanisole, and 2.1% (*v*/*v*) ethanedithiol) at room temperature for 4 h cleaved the crude ethyl ester intermediate from the resin and removed the side chain Pbf and ^t^Bu protecting groups as well. Following the concentration of the cleavage filtrate and the precipitation in cold diethyl ether of the crude ethyl ester intermediate (34% pure per analysis with RP-HPLC on an analytical C18 column (0.46 × 25 cm, 5 μm)), it was purified by RP-HPLC on a semi-preparative C18 column (1 × 25 cm, 5 μm). The column was eluted with a gradient of ddH_2_O containing 0.05% (*v*/*v*) TFA (mobile phase A) and acetonitrile containing 0.05% (*v*/*v*) TFA (mobile phase B) (0%–60% B in 60 min) at 4.5 mL/min and monitored at 214 nm. The pooled desired HPLC fractions were concentrated in vacuo to remove acetonitrile, and the remaining aqueous solution was lyophilized to afford the purified ethyl ester intermediate in an overall synthetic yield of 41% as a puffy white solid whose exact mass was confirmed by a unit-resolution ESI-MS analysis. This purified intermediate was then dissolved in a mixture of MeOH/ddH_2_O (3/1, *v/v*), and to the resulting solution was added at 0 °C LiOH to a final concentration of ~12.5 M. The reaction mixture was subsequently stirred at 4 °C overnight, acidified at 0 °C with 6 N HCl to pH ~1, and concentrated under reduced pressure. The ethyl ester hydrolysis product **4** was then isolated as a puffy white solid from the resulting residue by semi-preparative RP-HPLC as described above, using the following gradient of the afore-mentioned mobile phases A and B: 0%–40% B in 60 min. The purity of the purified **4** was >95% as verified by RP-HPLC on an analytical C18 column (0.46 × 25 cm, 5 μm) eluted with the following gradient of the afore-mentioned mobile phases A and B: 0%–30% B in 60 min. The exact mass of the purified compound **4** was confirmed by HRMS analysis (see Table 1).

### 3.3. Synthesis of ***5*** (Scheme 2)

This compound was prepared in the same manner as that of compound **4** (see above), with the exception of the lack of incorporation of two glycine residues in compound **5**. The crude **5** and the corresponding ethyl ester intermediate were also purified by semi-preparative RP-HPLC as described above, using the same respective gradients of mobile phases A and B (see above). Of note, the purified ethyl ester intermediate was obtained in an overall synthetic yield of 38% from its crude (31% pure per RP-HPLC analysis on an analytical C18 column (0.46 × 25 cm, 5 μm)). The purified **5** was also >95% pure based on RP-HPLC analysis on an analytical C18 column (0.46 × 25 cm, 5 μm) eluted with the same gradient of mobile phases A and B as that for the purified **4** (see above). The exact mass of the purified **5** was also confirmed by HRMS analysis (see Table 1).

### 3.4. Synthesis of ***6*** (Scheme 3)

This synthesis followed the standard Fmoc chemistry-based manual SPPS described above. The orthogonal deprotection of the Mtt protecting group on lysine side chain and the ensuing reaction of the exposed free amino group with ethyl 3-isothiocyanatopropionate, as well as the solution phase LiOH treatment were performed in the same manner as that described above for the synthesis of compound **4**. The crude **6** and the corresponding ethyl ester intermediate were also purified with semi-preparative RP-HPLC as described above, using the same respective gradients of mobile phases A and B (see above). The purified **6** was also >95% pure based on RP-HPLC analysis on an analytical C18 column (0.46 × 25 cm, 5 μm) eluted with the same gradient of mobile phases A and B as that for the purified **4** (see above). The exact mass of the purified **6** was confirmed by a unit-resolution ESI-MS analysis.

### 3.5. In Vitro Sirtuin Inhibition Assay

The HPLC-based sirtuin inhibition assay that our laboratory has been using over past several years was employed in the current study and was performed as described previously [37]. An assay solution (50 μL) contained the following components: 50 mM Hepes (pH 8.0), 137 mM NaCl, 2.7 mM KCl, 1 mM MgCl_2_, 1 mM DTT, β-NAD^+^ (0.5 mM for the SIRT1 and SIRT2 assays, 3.5 mM for the SIRT3 assay, 0.8 mM for the SIRT5 assay, or 0.2 mM for the SIRT6 assay), the peptide substrate (0.3 mM of the above-mentioned SIRT1/2/3 substrate for the SIRT1 assay, 0.39 mM of the above-mentioned SIRT1/2/3 substrate for the SIRT2 assay, 0.105 mM of the above-mentioned SIRT1/2/3 substrate for the SIRT3 assay, 0.88 mM of the above-mentioned SIRT5 substrate, or 0.02 mM of the above-mentioned SIRT6 substrate), one test compound (**4**, **5**, or **6**) with varied concentrations including 0, and a sirtuin (His_6_-SIRT1 or GST-SIRT1, 320 nM; His_6_-SIRT2, 309 nM; His_6_-SIRT3, 320 nM; GST-SIRT5, 370 nM; or His_6_-SIRT6, 313 nM). Of note, the same [S]/Km ratios for both substrates (~3.2 for the peptide substrates and ~5.6 for β-NAD^+^) were employed for the inhibition assays with all the five human sirtuins (SIRT1/2/3/5/6) employed in the current study. An enzymatic reaction was initiated by the addition of a sirtuin at 37 °C and was allowed to be incubated at 37 °C for 10 min (for the SIRT1 assay), 12 min (for the SIRT2 assay), 10 min (for the SIRT3 assay), 5 min (for the SIRT5 assay), or 12 min (for the SIRT6 assay) until quenched with the following stop solution: 100 mM HCl and 0.16 M acetic acid. A quenched assay solution was then centrifuged and the supernatant was injected into a reversed-phase C18 column (0.46 × 25 cm, 5 μm), eluting with a gradient of the afore-mentioned mobile phases A and B (0%–30% B in 40 min for the SIRT1/2/3 assays, 0%–30% B in 60 min for the SIRT5 assay, 0%–80% B in 60 min for the SIRT6 assay) at 1 mL/min, and UV monitoring at 214 nm. Turnover of the limiting substrate was maintained at <10%. Stock solutions of the test compounds were all prepared in ddH_2_O. IC_50_ values were estimated from the Dixon plots (1/*v*_0_ *v*s. (inhibitor)) [38] as an indication of the inhibitory potency.

### 3.6. Pronase Digestion Assay

This assay was also performed as described previously by our laboratory [37]. Fifty (50) μL of a solution of a test compound (**5**, **6**, or the control pentapeptide) in ddH_2_O (160 μM) was mixed thoroughly with 50 μL of a pronase solution in 100 mM Tris·HCl (pH 7.3) (8 ng/μL), and the resulting solution was incubated at 37 °C until quenched with a solution of acetic acid in ddH_2_O (1.0 M) at 0, 1.5, 3, and 6 min (for the control) or at 0, 7.5, 15, 30, and 60 min (for **5** and **6**). At each time point, 20 μL of a pronase digestion mixture was taken and treated with 40 μL of the 1.0 M acetic acid solution, and the whole mixture was vigorously vortexed, centrifuged, and the supernatant was injected into a reversed-phase C18 analytical HPLC column (0.46 × 25 cm, 5 μm). The C18 column was eluted with a gradient of the afore-mentioned mobile phases A and B (0%–30% B in 40 min for the assay with the control pentapeptide, 0%–40% B in 60 min for the assays with **5** and **6**) at 1 mL/min and with UV monitoring at 214 nm. The HPLC peak areas obtained for a given test compound at different time points were used to estimate the percentage remaining for this test compound with the digestion time. The graph of the percentage remaining versus time was used to compare the proteolytic stability of different test compounds, as shown in Figure 4.

## 4. Conclusions

In the current study, we discovered that compound **5**, a side chain-to-side chain cyclic pentapeptide harboring the SIRT5 inhibitory warhead *N*^ε^-carboxyethyl-thiocarbamoyl-lysine at its central position, behaved as a strong and selective (versus human SIRT1/2/3/6) inhibitor against human SIRT5-catalyzed deacylation reaction. This compound was also found to be proteolytically much more stable than its linear counterpart **6**. Despite being a comparably strong SIRT5 inhibitor to the linear peptidic compounds **1** and **2** (the strongest and most selective SIRT5 inhibitors reported in the current literature), the cyclic peptide-based compound **5** would be at a better position to serve as a lead for the development of stronger, selective, metabolically-stable, and cell-permeable SIRT5 inhibitors. We are currently endeavoring to construct a macrocycle bridging unit combinatory library and to incorporate the library members into the basic structural scaffold of compound **5**, furnishing a library of the analogs of compound **5**. By so doing, we hope the relevant chemical space at different sirtuin active sites could be more comprehensively explored, and stronger and selective cyclic peptide-based SIRT5 inhibitors also harboring the SIRT5 inhibitory warhead N^ε^-carboxyethyl-thiocarbamoyl-lysine could be found.

## Figures and Tables

**Table 1:** HRMS analysis of compounds **4** and **5** ^a^.

Compound	Ionic Formula	Calculated *m*/*z*	Observed *m*/*z*
**4**	[C_46_H_82_N_15_O_13_S]^+^	1084.5932	1084.5922
**5**	[C_42_H_76_N_13_O_11_S]^+^	970.5502	970.5498

^a^ Both compounds were measured with the positive ion mode of electrospray ionization.

**Table 2:** Sirtuin inhibition by compounds **4**–**6** ^a^.

Compound	IC_50_ (μM) ^b^				
	SIRT5	SIRT2	SIRT1	SIRT3	SIRT6
**4**	6.0 ± 3.0	9.2 ± 0.57	n.d. ^c^	n.d.	n.d.
**5**	7.5 ± 4.0	>1000	>1000	>200	>200
**6**	7.6 ± 1.5	96.4 ± 18.5	>1000	>1000	>1000

^a^ See “Experimental Section” for the sirtuin inhibition assay details. The IC_50_ values for compound **5** are bold and underlined to highlight its selective inhibition against SIRT5 versus SIRT1/2/3/6; ^b^ IC_50_, the inhibitor concentration at which an enzymatic reaction velocity is reduced by 50%; ^c^ n.d., not determined in the current study.

## Abbreviations

ADP	adenosine diphosphate
β-NAD^+^	β-nicotinamide adenine dinucleotide
NAM	nicotinamide
2′-*O*-AADPR	2′-*O*-acyl-ADP-ribose
IC_50_	the inhibitor concentration at which an enzymatic reaction velocity is reduced by 50%
Ki	inhibition constant
SPPS	solid phase peptide synthesis
MBHA	4-methylbenzhydrylamine
TFA	trifluoroacetic acid
DMF	*N*,*N*-dimethylformamide
RP-HPLC	reversed-phase high performance liquid chromatography
HRMS	high-resolution mass spectrometry
HBTU	2-(1*H*-benzotriazole-1-yl)-1,1,3,3-tetramethylaminium hexafluorophosphate
HOBt	*N*-hydroxybenzotriazole
NMM	*N*-methylmorpholine
Km	The substrate concentration at which an enzymatic reaction velocity is half-maximal
